# Insulin-like growth factor-1 rescues synaptic and motor deficits in a mouse model of autism and developmental delay

**DOI:** 10.1186/2040-2392-4-9

**Published:** 2013-04-27

**Authors:** Ozlem Bozdagi, Teresa Tavassoli, Joseph D Buxbaum

**Affiliations:** 1 Seaver Autism Center for Research and Treatment, Icahn School of Medicine at Mount Sinai, New York, NY, USA; 2 Department of Psychiatry, Icahn School of Medicine at Mount Sinai, New York, NY, USA; 3 Department of Neuroscience, Icahn School of Medicine at Mount Sinai, New York, NY, USA; 4 Department of Genetics and Genomics Sciences, Icahn School of Medicine at Mount Sinai, New York, NY, USA; 5 Friedman Brain Institute, Icahn School of Medicine at Mount Sinai, New York, NY, USA; 6 Mindich Child Health and Development Institute, Icahn School of Medicine at Mount Sinai, New York, NY, USA

**Keywords:** Pharmacotherapy, Personalized medicine, Individualized medicine, 22q13 deletion syndrome, Phelan-McDermid syndrome

## Abstract

**Background:**

Haploinsufficiency of *SHANK3*, due to either hemizygous gene deletion (termed 22q13 deletion syndrome or Phelan-McDermid syndrome) or to gene mutation, accounts for about 0.5% of the cases of autism spectrum disorder (ASD) and/or developmental delay, and there is evidence for a wider role for SHANK3 and glutamate signaling abnormalities in ASD and related conditions. Therapeutic approaches that reverse deficits in *SHANK3*-haploinsufficiency may therefore be broadly beneficial in ASD and in developmental delay.

**Findings:**

We observed that daily intraperitoneal injections of human insulin-like growth factor 1 (IGF-1) over a 2-week period reversed deficits in hippocampal α-amino-3-hydroxy-5-methyl-4-isoxazolepropionic acid (AMPA) signaling, long-term potentiation (LTP), and motor performance that we had previously reported in *Shank3*-deficient mice. Positive effects were observed with an IGF-1 peptide derivative as well.

**Conclusions:**

We observed significant beneficial effects of IGF-1 in a mouse model of ASD and of developmental delay. Studies in mouse and human neuronal models of Rett syndrome also show benefits with IGF-1, raising the possibility that this compound may have benefits broadly in ASD and related conditions, even with differing molecular etiology. Given the extensive safety data for IGF-1 in children with short stature due to primary IGF-1 deficiency, IGF-1 is an attractive candidate for controlled clinical trials in *SHANK3*-deficiency and in ASD.

## Findings

SHANK proteins are master scaffolding proteins of the postsynaptic density (PSD) of glutamatergic synapses and are critical determinants of glutamate transmission and synaptic spine dynamics [1]. Loss of one functional copy of *SHANK3* accounts for about 0.5% of the cases of autism spectrum disorder (ASD) and/or developmental delay [2], and there is likely a wider role for SHANK3 and glutamate signaling abnormalities in ASD and related neurodevelopmental disorders [3,4]. Targeted disruption of the full-length form of *Shank3* (sometimes called Shank3a) in mice leads to deficits in hippocampal AMPA signaling, long-term potentiation (LTP), and motor performance [5-7], likely reflecting delayed synaptic development as shown by the reduced AMPA signaling [5] and decreased levels of PSD-95 (unpublished results). IGF-1, which enters the central nervous system (CNS) through an interaction with lipoprotein-related receptor 1 (LRP1) [8], has multiple effects on neuronal and synaptic development and function, including effects on neurogenesis and synaptogenesis [9]. IGF-1 treatment also enhances the PSD as measured both by PSD length and by levels of PSD-95 [10,11]. Recombinant human IGF-1 has substantial human safety data and is approved for use in children, making IGF-1 an attractive compound for evaluation in neurodevelopmental disorders.

**Figure 1:**
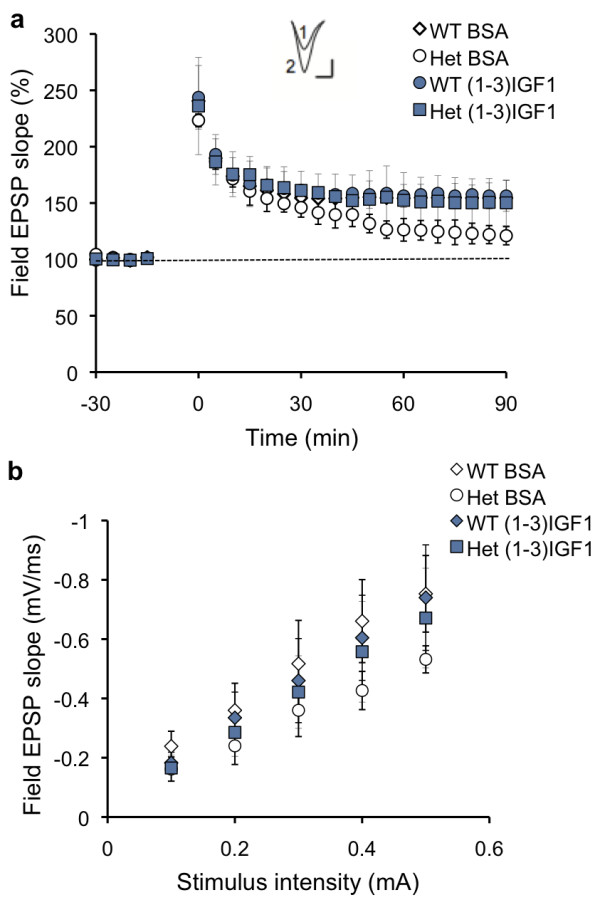
**(1–3)IGF-1 reverses deficits in LTP and basal synaptic properties in *Shank3*-deficient mice.** Wild-type (WT) and heterozygous (Het) mice were treated with saline or (1–3)IGF-1 for 2 weeks before testing (injections began at postnatal day (PND) 13 to 15 and animals were analyzed immediately after the last injection). Methods for all experiments were as described previously [5], with 3 to 4 mice per group, and 1 to 2 slices per animal. (**a**) Hippocampal LTP was induced with high-frequency stimulation. Inset: Representative excitatory postsynaptic potential traces at 90 min after LTP induction from saline-injected (1) and (1–3)IGF-1-injected (2) heterozygous mice (scale bar: 0.5 mV, 10 ms). (**b**) Input–output curves comparing field excitatory postsynaptic potential (EPSP) slopes (mV/ms) as a function of stimulation intensity (mA). EPSP: excitatory postsynaptic potential; Het: heterozygous; LTP: long-term potentiation; PND: postnatal day; WT: wild-type.

**Figure 2:**
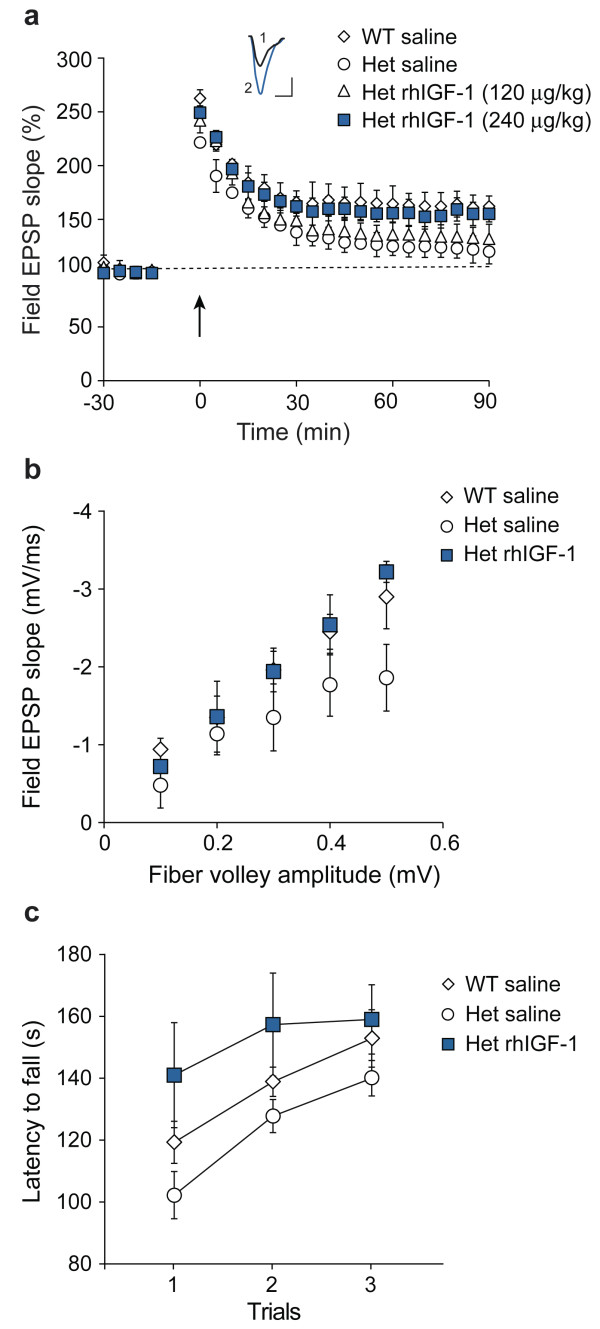
**IGF-1 reverses deficits in LTP, AMPA signaling, and motor function in *Shank3*-deficient mice.** Wild-type (WT) and heterozygous (Het) mice were treated with saline or recombinant human IGF-1 (rhIGF-1) for 2 weeks (beginning at PND 13 to 15) before testing and analyzed immediately after the last injection. Methods for all experiments were as described previously [5,7], with 4 to 9 mice per group. (**a**) Hippocampal LTP was induced with high-frequency stimulation. Inset: Representative excitatory postsynaptic potential traces at 90 min after LTP induction from saline-injected (1) and rhIGF-1-injected (2) heterozygous mice (scale bar: 0.5 mV, 10 ms). (**b**) Slices were incubated in the presence of the N-Methyl-D-aspartate (NMDA) antagonist R-2-amino-5-phosphonopentanoate (APV) to expose AMPA receptor signaling. (**c**) Mice were tested for motor performance and motor learning by measuring latencies to fall off a rotating rod over three trials. Het: heterozygous; LTP: long-term potentiation; NMDA: N-Methyl-D-aspartate; rhIGF-1: recombinant human IGF-1; WT: wild-type.

To investigate whether IGF-1 could reverse deficits in a preclinical model of *SHANK3*-haploinsufficiency, we made use of a mouse with hemizygous loss of full-length *Shank3* due to targeted disruption of the ankyrin repeat domain (ARD) [5]. This isoform has been directly implicated in ASD, language delay, and intellectual disability (ID), as there exist disruptive *de novo* point mutations in ARD in patients with ASD and ID [12,13]. In all studies, we compared heterozygous mice with wild-type littermates using heterozygote × heterozygote mating. Consistent with previous results from our group [5], LTP induced by high-frequency stimulation was reduced in the heterozygous mice compared to wild-type littermates in the current experiments (Figures 1a and 2a) (for example, in Figure 2a, repeated measures ANOVA was used for analysis of the last five time points, *F*(1,6) = 33.71, *P* = 0.001).

We first tested an active peptide derivative of IGF-1, (1–3)IGF-1, which has been shown to cross the blood–brain barrier and rescue Rett syndrome symptoms in *Mecp2*-deficient mice [11]. We observed that intraperitoneal injections at 10 μg/g/day for 2 weeks restored normal hippocampal LTP in *Shank3* heterozygous mice but had no effect on wild-type mice (repeated measures ANOVA was used to analyze the last five time points, *F*(3,11) = 6.07, *P* = 0.011). In *post hoc* analyses, vehicle-treated heterozygous mice were significantly different from wild-type mice (*P* = 0.004), while (1–3)IGF-1 treated heterozygous mice were not (*P* = 0.66). Furthermore, peptide treatment reversed deficits in the mean slope of the input/output (I/O) function (Figure 1b) (one-way ANOVA, *F*(3,19) = 4.25, *P* = 0.02). Vehicle-treated heterozygous mice were significantly different from vehicle-treated wild-type mice (*P* = 0.001), while (1–3)IGF-1 treated heterozygous mice were not different from vehicle-treated wild-type (*P* = 0.89), and there were no significant differences between vehicle-treated wild-type mice and wild-type mice treated with IGF-1 (*P* = 0.812), so further studies used just three conditions.

We next administered full-length IGF-1, like that used in children with short stature due to primary IGF-1 deficiency, by intraperitoneal injection at 240 μg/kg/day, starting at PND 13 to 15 and continuing for 2 weeks (Figure 2a). This dose, chosen because it represents the maximum dose according to the current FDA label for IGF-1, was effective in rescuing deficits in LTP (repeated measures ANOVA was used to analyze the last five time points, comparing heterozygous mice with and without IGF-1, *F*(1,6)=28.04, *P*=0.002). In contrast, lower dose IGF-1 (120 μg/kg/day for 2 weeks) was associated with more modest reversal of deficits in LTP (for the last five time points: *F*(1,6)=2.62, *P*=0.012), showing a dose–response effect and providing preclinical dosing information.

Specific deficits in the glutamate AMPA receptor component of neural signaling [5] were also reversed by a 2-week treatment of 240 μg/kg/day full-length IGF-1 (Figure 2b). The mean slope of the I/O function was 0.50 ± 0.14 for wild-type, 0.34 ± 0.06 for *Shank3* heterozygotes and 0.61 ± 0.059 for IGF-1 injected heterozygotes (one-way ANOVA, *F*(2,9) = 8.62, *P* = 0.008). In *post hoc* analyses, vehicle-treated heterozygous mice were significantly different from vehicle-treated wild-type mice (*P* = 0.039), while IGF-1-treated heterozygous mice were not different from vehicle-treated wild-type mice (*P* = 0.12).

Patients with *SHANK3*-haploinsufficiency frequently present with hypotonia and motor deficits of variable severity, and we have observed subtle motor deficits in *Shank3*-heterozygous mice [5,7]. After treating male heterozygous mice with 240 μg/kg/day for 2 weeks, we observed enhanced motor performance following treatment (Figure 2c) (*F*(2,20) = 3.98, *P* = 0.03).

Our results provide preclinical evidence for a beneficial role for IGF-1 in *SHANK3*-haploinsufficiency. Moreover, as there is emerging evidence that the SHANK3 pathway and the postsynaptic density, which it helps sculpt, play a role in many neurodevelopmental disorders, as evidenced by large-scale genetic, proteomic, and gene expression studies [3,4,14], therapies for *SHANK3* deficiency and synaptic development represent important targets that could have a widespread positive impact for neurodevelopmental disorders. The beneficial effects of IGF-1 in models of Rett syndrome [11,15] are consistent with this hypothesis.

There are some limitations to the current study. We, and others working with similar *Shank3*-deficient mice, see only limited behavioral abnormalities, with none except for rotarod deficits at the ages where we carried out the IGF-1 treatments and electrophysiological studies. For this reason, the phenotypes we measure are somewhat limited. In addition, a mechanistic understanding of the neuronal effects of IGF-1 has eluded the neuroscience community and we cannot precisely explain how IGF-1 reverses the deficits observed. We do hope, however, that our findings, together with those on IGF-1 in Rett syndrome models, may help spur further research on the action of IGF-1 in the CNS. We did not see any effect produced by the (1–3)IGF-1 peptide on control animals but we did not test the effects of full-length IGF-1 on wild-type mice. There could be enhanced LTP or rotarod performance in control animals following treatment with full-length IGF-1. Many drugs have effects on both healthy and non-healthy individuals and there is hence no *a priori* reason to assume that IGF-1 has no effect on control animals. In fact, given the positive effects of IGF-1 in Rett syndrome models it is likely that IGF-1 has a general effect on CNS function, which might also be observed in controls.

In summary, our results show that IGF-1, approved for use in children, can lead to functional improvements in a mouse model of ASD and developmental delay, representing an important preclinical step towards novel therapeutics. Clinical trials of IGF-1 in *SHANK3*-deficient individuals and in ASD are now underway (ClinicalTrials.gov Identifier NCT01525901).

## Abbreviations

ARD: Ankyrin repeat domain; ASD: Autism spectrum disorder; AMPA: α-Amino-3-hydroxy-5-methyl-4-isoxazolepropionic acid; CNS: Central nervous system; EPSP: Excitatory postsynaptic potential; Het: Heterozygous; ID: Intellectual disability; IGF-1: Insulin-like growth factor 1; I/O: Input/output; LRP1: Lipoprotein-related receptor 1; LTP: Long-term potentiation; NMDA: N-Methyl-D-aspartate; PND: Postnatal day; PSD: Postsynaptic density; rhIGF-1: Recombinant human IGF-1; WT: Wild-type.

## Competing interests

OB and JDB have submitted a patent on this work.

## Authors’ contributions

OB and JDB designed the experiments, interpreted the results, and prepared the manuscript. OB carried out all electrophysiological and behavioral studies. TT analyzed the results and help in aspects of experimental design. All authors read and approved the final manuscript.
